# Mental health resources and its equity in Central South of China: A case study of Hunan Province

**DOI:** 10.1371/journal.pone.0272073

**Published:** 2022-10-12

**Authors:** Bang-An Luo, Sheng Li, Si Chen, Lu-Lu Qin, Yi-Wei Chen, Man-Ling Shu, Xin-Yi Liao

**Affiliations:** 1 Department of Social Medicine and Health Management, Xiangya School of Public Health, Central South University, Changsha, China; 2 Department of Mental Health, Brain Hospital of Hunan Province, Changsha, Hunan, China; 3 Department of Social Medicine and Health Management, School of Medicine, Hunan Normal University, Changsha, China; 4 Department of Neurology, Xiangya Third Hospital, Central South University, Changsha, China; International School, Vietnam National University, VIET NAM

## Abstract

**Objectives:**

Mental health resources are an important basis for coping with mental health services. The equity is an important index of a reasonable allocation of health resources. This study aims to evaluate the mental health resources and its equity allocation in Hunan Province, which is one of the typical central south areas of China, so as to provide reference for the development of mental health in China and other areas.

**Methods:**

Data related to mental health resources was obtained from the Project of Mental Health Resources in Hunan Province, which was conducted by the Department of Hunan Mental Health Center in 2019. The Gini coefficient, the Theil index and other indicators were employed to quantitatively evaluate the equity of mental health resources’ allocation.

**Results:**

By the end of 2018, there were a total of 141 mental health institutions in Hunan Province of China, the bed density was 5.31 beds per 10,000 people, the ratio of doctors to nurses was 2.20, the number of outpatients of mental health institutions was 1288,047 per year. The mental health resources’ allocation in terms of demographic dimension were in a preferred status with the Gini values all less than 0.3, and the Gini values for mental health resources`allocation in terms of geographical dimension ranged from 0.24 to 0.35. The Theil index for mental health allocation in terms of demographic dimension was lower than 0.05, and the Theil index for mental health allocation in terms of geographical dimension ranged from 0.04 to 0.11.

**Conclusions:**

The shortage of mental health resources is still the priority issue to be increased and optimized by policy-makers in Hunan in the future, especially the human resources. Moreover, the utilization of mental health resources was low though its equity was fair. Policy-makers need to consider the high utilization and geographical accessibility of health resources among different regions to ensure people in different regions could get access to available health services.

## Introduction

Mental disorders are a major public health problem and severe social problem that affect global economic development. The incidence rates of mental disorders are on the rise following the rapid transformation of economic growth and social structures worldwide. In China, the lifetime incidence rate, annual incidence rate and incidence rate of various mental disorders during 2012–2015 were 16.6%, 16.6% and 9.3% respectively [1]. The disease burden of mental disorders in 2016 accounted for 13% of burden from non-infectious diseases in China and for 17% of burden from mental diseases globally [2–4]. In the USA, about 57.7 million people suffered from mental disorders annually, and 1/17 of them had severe mental health problems [5]. Hence, the economic burdens of mental disorders are huge. The indirect expenses of mental diseases in the USA were estimated to exceed 79 billion dollars, of which about 63 billion dollars reflected the losses of productivity due to diseases [6]. In the European Union, mental health problems account for 3% to 4% of gross national product [7]. In the meantime, the requirements for health levels and the demands for mental health services are increasing. Then, mental health resources are an important basis for coping with mental health services.

However, the mental health service system is still encountered with many shortcomings, such as shortage of resources, low rate of mental disease therapy, incompleteness of the mental health institution network service system, and wasting of mental health resources due to unreasonable resource allocation. About 30% of the global population is affected by mental disorders every year, and nearly two-thirds of them cannot get the therapy in need [8]. The availability of psychiatrists is less than 1/100000 in most parts of Southeast Asia and less than 1/1million in Sub-Saharan, and only 2% of the national budget is used for mental health globally, according to World Health Organization (WHO) [9]. The availability rate of psychiatrists in China is 1.55/100000, which is lower than the global average rate of 3.96/100000 [10]. The availability rates of psychiatrists per 100,000 people in India, Pakistan, Nigeria and Ethiopia are 0.301, 0.185, 0.06 and 0.04 respectively [11]. Such uneven geographical distribution of limited mental health resources further decreases the opportunity of mental health care. For instance, most psychiatrists in low- and middle- income countries are working in the centers of main cities, but due to traffic problems, rural people are unable to obtain their services [12,13]. A WHO survey on 113 countries shows that mental health resource distributions are largely different among hospitals and communities, as specialized mental hospitals possess 64% resource investments, and general hospitals occupy 21% investments, but less than 16% of resources are directed to local facilities and communities [14,15]. Given the difficulty in improving the service abilities of mental health institutions caused by the shortage and uneven distribution of mental health resources, analyzing mental health resource and its distribution is very necessary.

In China, the development of the mental health system has attracted increasing attention. Hunan province, has paid more and more attention to mental health in recent years, especially since the implementation of the National Continuing Management and Intervention Program for Psychoses (also named 686 Program) in 2004. Hunan province, as a typical south-central province in China, its geographical position is from 24°38′ to 30°08′N latitude and from 108°47′to 114°15′E longitude. The total population of Hunan was 73.3 million in 2018, with the gross domestic product (GDP) ranked 8th among the provinces in China (8/34). Among the GDP, the proportion of agriculture, industry and services is 8.5%, 39.7% and 51.8%, which was similar to other areas in China.

According to previous studies, the lifetime prevalence rate of Severe Mental Illness in Hunan was increasing, which ascended from 5.690‰ (1982), 6.550‰ (1993) to 10.100‰(2014) [16,17]. Moreover, a previous study showed that the resources of mental health institutions in Hunan Province were relatively poor in 2002. However, there is still a lack of literature report on the research of mental health services and its equity allocation in Hunan Province. Therefore, there is an urgent issue to assess the resources of mental health in Hunan Province and its equity after more than ten years of development. Thus, this study aims to evaluate the mental health resources and its equity allocation in Hunan Province, which is one of the typical central south areas of China, so as to provide reference for the development of mental health in China and other areas.

## Materials and methods

### Ethical approval

The study was conducted in accordance with the Declaration of Helsinki. Ethical approval was obtained from the Research Ethics Committee of the Brain Hospital of Hunan Province on December 16 of 2018 (NO.20181216).

### Data sources

There are 13 prefecture-level cities and 1 autonomous prefecture in Hunan Province, China. Data related to mental health resources was obtained from the Project of Mental Health Resources in Hunan Province, which was conducted by the Department of Hunan Mental Health Center in 2019. The data of this project is originally collected by the staff responsible for mental health resources investigation, which is further audited and managed by the Department of Hunan Mental Health Center. The administrative permission from the Department of Hunan Mental Health Center is required to access and use the data in this system, and we have been permitted to use these data for analysis in this study.

The number of annual patients and information related to mental health resources (including human resources, diagnosis and treatment, number of beds and so on) of Hunan Province in 2018 were collected. The number of annual patients reported was obtained by one of our researchers who had the access to identifying related information in a fully anonymized and de-identified manner, which was approved by IRB prior to the data use.

### Gini coefficient

The Gini coefficient is regarded as one of the superior tools for evaluating the equity of health resources allocation. The range of Gini coefficient is 0–1, and the closer the Gini coefficient is to 0, the higher the equity of resource allocation is, and the closer the Gini coefficient is to l, which means that the resource allocation is more unfair [18–20]. This study refers to the standard in economics, that is, the Gini coefficient below 0.2 is regarded as absolutely fair, 0.2–0.3 as relatively fair, 0.3–0.4 as relatively reasonable, 0.4–0.5 as a large gap. 0.4 is often regarded as the warning line of the gap when the Gini coefficient is above 0.5. The Gini coefficient formula used in this study is as follows:

$$G=\sum_{i=1}^{n-1}(X_{i}Y_{i+1}-X_{i+1}Y_{i})$$

Where G is the Gini coefficient and represents the cumulative percentage of the cumulative population (geographical area) and the cumulative percentage of evaluated health resources (medical and health institutions, beds, health personnel) respectively.

### Theil index

The Theil index is also a common statistical index to reflect the fair degree of resource allocation, with a value range of 0–1. The lower the value, the smaller the difference in resource allocation, and the equity is relatively better. The Theil index mainly considers the influence of population factors on the equity of health resource allocation, interpreting the measurement of medical resource gap as the amount of information contained in the information that converts population share into resource share [21–24]. It is sensitive to the change of resource allocation efficiency, has good decomposition property, and can find out the equity and contribution of different levels and groups, which is an ideal analysis tool for unfair decomposition [25]. The Theil index formula used in this study is as follows:

$$T=\sum_{i=1}^{\text{n}}P_{i}\;\text{log}\;\frac{P_{i}}{Y_{i}}$$

The above formula is the total Theil index for the allocation of mental health resources, in which the population (geographical area) of each city (autonomous prefecture) accounts for the proportion of the total population (geographical area) of the whole province. That is, mental health resources owned by each city (autonomous prefecture) in the total amount of the whole province. N represents the number of research areas (this study is the cities and autonomous prefectures of Hunan Province).

### Statistical analysis

Epidata3.0 was used to enter data twice independently and check for errors to improve accuracy, and data was analyzed with SPSS 20.0 (SPSS/IBM, Armonk, New York, USA). Descriptive methods were applied to analyze the status quo of mental health service. The Gini coefficient and Theil index were used to measure the equality of distribution per 10000 people and per square kilometer of institutions, doctors, beds and nurses. The quantity of mental health resources by every 10000 people (or in 1 square kilometers) in each city was calculated based on the number of permanent population (or geographical area) and the numbers of mental health institutions, doctors, nurses and beds in each city. The definition of equity indicators is shown in Table 1.

**Table 1:** Definition of equity indicators.

Variables	Definition	Sources
Number of mental health institutions	At the end of the year, the sum number of mental health institutions.	The Project of Mental Health Resources in Hunan Province
Number of beds	The practical beds in mental health institutions (also referred to the number of actual beds).	The Project of Mental Health Resources in Hunan Province
Number of workers	At the end of the year, the sum number of doctors, nurses, mental therapists, mental consultants, rehabilitation therapists,social workers, and public health doctors in mental health institutions who have obtained registration certificates and are actually engaged in disease prevention and control, medical treatment, or mental health care.	The Project of Mental Health Resources in Hunan Province
Number of doctors	At the end of the year, The sum number of medical practitioners and assistant doctors engaged in mental health diagnosis and treatment in mental health institutions.	The Project of Mental Health Resources in Hunan Province
Number of outpatients	At the end of the year, the sum number of psychiatric outpatients	The Department of Hunan Mental Health Center
Other institutional levels	Mental health institutions that are graded without the approval of the health audit department	The Project of Mental Health Resources in Hunan Province
Other Category	The sum of mental health institutions other than mental hospitals, psychiatric departments of general hospitals, psychiatric departments of traditional Chinese medicine Hospitals and mental rehabilitation institutions	The Project of Mental Health Resources in Hunan Province, Hunan statistical yearbook 2018

## Results

### Mental health resources of Hunan province

#### General information of mental health institutions

By the end of 2018, there were 141 mental health institutions in Hunan Province of China. Among them, 89 were psychiatric hospitals (28 primary hospitals and 45 secondary hospitals), accounting for 63.1%. There were 42 psychiatric departments in general hospitals (13 first-class hospitals and 18 second-class hospitals), accounting for 29.8% (Table 2).

**Table 2:** General information of mental health institutions.

Institutional level	Category					Total
	Psychiatric specialist hospital	Department of Psychiatry, General Hospital	Department of Psychiatry, Traditional Chinese Medicine Hospital	Mental rehabilitation institution	Other ^②^	
First-class hospital	28	13	-	-	1	42
Second-level hospital	45	18	6	1	-	70
Tertiary hospital	-	10	-	-	-	10
Other ^①^	16	1	-	1	1	19
Total	89	42	6	2	2	141

Note: 1 unclassified institution; 2 mental health institutions except psychiatric hospitals, psychiatric departments of general hospitals, psychiatric departments of traditional Chinese medicine hospitals and mental rehabilitation institutions.

#### The number of beds in mental health institutions

By the end of 2018, Hunan Province has a total resident population of 73.2662 million, a total of 25554 beds in mental health institutions, 38911 beds are actually open, and the bed density is 5.31 beds per 10,000 people, slightly higher than the national average. According to the bed density distribution map, the bed density is the highest in Changsha area and the lowest in Yongzhou area (Table 3).

**Table 3:** General information of mental health resources in Hunan Province in 2018.

City	Population (/10000)	institutions	beds	Actual beds	doctors	nurses	Psychotherapist	Psychological counselor	Rehabilitation specialist	Social worker	Public Health physician	Doctor density (/100000)	Nurse density (/100000)	Doctor-to-nurse ratio	Bed ratio	Number of outpatients	Per capital number of outpatients per doctor
Changsha	728.86	15	6585	8808	340	837	10	31	23	29	13	4.66	11.48	1:2.5	1:0.1	329556	2.66
Zhuzhou	402.68	10	1226	1702	102	220	6	25	0	0	4	2.53	5.46	1:2.2	1:0.1	113269	3.04
Xiangtan	288.8	7	695	1576	108	278	1	5	0	11	1	3.74	9.63	1:2.6	1:0.2	50385	1.28
Yueyang	568.7	8	1635	2748	182	365	10	14	15	18	10	3.20	6.42	1:2.0	1:0.1	103552	1.56
Yiyang	477.5	7	920	1932	141	147	3	11	0	12	11	2.95	3.08	1:1.0	1:0.1	80042	1.56
Shaoyang	828.28	17	2927	4195	242	463	10	31	14	14	7	2.92	5.59	1:1.9	1:0.1	105359	1.19
Huaihua	523.52	11	1654	2052	125	299	8	14	6	11	7	2.39	5.71	1:2.4	1:0.1	59193	1.30
Changde	605.29	12	1607	2205	117	193	3	19	11	25	10	1.93	3.19	1:1.6	1:0.1	56115	1.31
Yongzhou	644.61	12	1416	2354	106	245	11	12	7	18	4	1.64	3.80	1:2.3	1:0.1	27755	0.72
Chenzhou	535.32	12	1647	2102	133	261	12	21	4	13	11	2.48	4.88	1:2.0	1:0.1	126086	2.60
Hengyang	800.53	12	2184	3098	200	476	5	11	7	24	8	2.50	5.95	1:2.4	1:0.2	99382	1.36
Loudi	455.17	11	2028	3369	176	319	3	8	4	10	11	3.87	7.01	1:1.8	1:0.1	54033	0.84
Zhangjiajie	170.13	2	260	730	32	67	0	5	0	0	0	1.88	3.94	1:2.1	1:0.1	9700	0.83
Xiangxi	297.24	5	720	2040	79	377	3	14	1	5	20	2.66	12.68	1:4.8	1:0.2	73620	2.55
Total	7326.62	141	25554	38911	2083	4547	85	221	92	190	117	2.84	6.21	1:2.2	1:0.1	1288047	1.69

#### General information of mental health human resources

By the end of 2018, Hunan harbored 7335 mental health workers, including doctors, nurses, mental therapists, mental consultants, rehabilitation therapists, social workers, and public health doctors. Of them, there were 2083 doctors and 4547 nurses, accounting for 28.40% and 61.99% of all mental health workers in Hunan respectively. The remaining 9.61% of the staff were other five types of professional workers, including 190 social workers (2.59%), 221 mental consultants (3.01%), 117 public health doctors (1.60%), 92 rehabilitation therapists (1.25%) and 85 mental therapists (1.16%). Within Hunan, Changsha harbors the largest share of 1283 mental health workers, but Zhangjiajie possesses the lowest share of 104 mental health workers, which account for 17.49% and 1.42% of mental health staff in Hunan respectively (Table 3).

From the perspective of population density, the densities of mental health doctors and nurses in Hunan are 2.84/100000 and 6.21/100000 respectively, which are both higher than the national levels in China. Particularly, the doctor densities of Changde and Yongzhou are 1.93/100000 and 1.64/100000 respectively, which are both lower than the national levels of China. The doctor to nurse ratio is 1:2.2, and the bed to nurse ratio is 1:0.1 in Hunan, which are higher than and lower than the corresponding national levels respectively (Table 3).

#### General information of diagnosis and therapy of mental health

The number of outpatient services by mental health institutions of Hunan in 2018 was 1288047 patients, and the daily number of outpatient services by doctors per capita was 1.70 patients. Among cities of Hunan, the number of outpatient services maximized in Changsha and minimized in Zhangjiajie. The daily number of outpatient services by doctors per capita maximized to 3.04 patients in Zhuzhou, followed by Changsha (2.66 patients), and minimized to 0.72 patient in Yongzhou (Table 3).

### Equity of mental health resource allocation

#### Equity allocation of mental health resources according to population and geographical areas

Of the 141 mental health institutions in 2018, the number of mental health institutions per 10000 people was 0.02. Particularly, the number of mental health institutions per 10000 people minimized to 0.01 in Zhangjiajie, Yueyang, Yiyang, and Hengyang, and was 0.02 in other cities. The average number of mental health beds per 10000 people was 5.31 in 2018 in Hunan, but maximized to 12.08 in Changsha, followed by Loudi (7.40), and minimized to 3.64 in Changde. The average number of mental health doctors per 10000 people in 2018 was 0.28 in Hunan, and maximized to 0.47 in Changsha, but minimized to 0.16 in Yongzhou. The average number of mental health nurses per 10000 people in 2018 was 0.62 in Hunan, and maximized to 1.27 in Xiangxi, followed by Changsha (1.15), but minimized to 0.31 in Yiyang (Table 4).

**Table 4:** Equity allocation of mental health resources according to population and geographical areas.

City	Number of configurations per 10,000 people				Number of configurations per square kilometer			
	institutions	beds	doctors	nurses	institutions	beds	doctors	nurses
Changsha	0.02	12.08	0.47	1.15	<0.01	0.75	0.03	0.07
Zhuzhou	0.02	4.23	0.25	0.55	<0.01	0.15	0.01	0.02
Xiangtan	0.02	5.46	0.37	0.96	<0.01	0.31	0.02	0.06
Yueyang	0.01	4.83	0.32	0.64	<0.01	0.18	0.01	0.02
Yiyang	0.01	4.05	0.30	0.31	<0.01	0.16	0.01	0.01
Shaoyang	0.02	5.06	0.29	0.56	<0.01	0.20	0.01	0.02
Huaihua	0.02	3.92	0.24	0.57	<0.01	0.07	<0.01	0.01
Changde	0.02	3.64	0.19	0.32	<0.01	0.12	0.01	0.01
Yongzhou	0.02	3.65	0.16	0.38	<0.01	0.10	<0.01	0.01
Chenzhou	0.02	3.93	0.25	0.49	<0.01	0.11	0.01	0.01
Hengyang	0.01	3.87	0.25	0.59	<0.01	0.20	0.01	0.03
Loudi	0.02	7.40	0.39	0.70	<0.01	0.42	0.02	0.04
Zhangjiajie	0.01	4.29	0.19	0.39	<0.01	0.08	<0.01	0.01
Xiangxi	0.02	6.86	0.27	1.27	<0.01	0.13	0.01	0.02
Total	0.02	5.31	0.28	0.62	<0.01	0.18	0.01	0.02

The average number of mental health institutions per square kilometers in 2018 was lower than 0.01 in Hunan. The average number of mental health beds per square kilometers was 0.18 in Hunan, and maximized to 0.75 in Changsha, but minimized to 0.07 in Huaihua. The average number of mental health doctors per square kilometers was 0.01 in Hunan, and maximized to 0.03 in Changsha, and minimized to <0.01 in Huaihua, Yongzhou and Zhangjiajie. The average number of mental health nurses per square kilometers was 0.02 in Hunan, and maximized to 0.07 in Changsha, and minimized to 0.01 in Zhangjiajie, Changde, Huaihua, Yongzhou, Yiyang, and Chenzhou (Table 4).

#### The Gini index of mental health institution resources

The Gini index of mental health institutions, beds, doctors and nurses of Hunan in 2018 ranged from 0.15 to 0.23, indicating the equity allocation of mental health resources in terms of population was equal. As for geographical allocations, the allocation of medical health institutions is equal, and the allocations of medical health beds, doctors and nurses are relatively equal. Generally, the equity of mental health resource allocation based on population is higher than that of geographical allocation (Table 5).

**Table 5:** The Gini coefficient of resource allocation of mental health institutions.

Resource category	Population allocation	Geographical allocation
Mental health institution	0.10	0.24
Beds	0.21	0.35
Doctors	0.16	0.33
Nurse	0.23	0.34

#### The Theil index of mental health institution resource allocation

The Theil index of mental health institutions, beds, doctors and nurses of Hunan in 2018 was 0.01, 0.04, 0.01 and 0.04 respectively. The overall situation is similar to that of Gini index, and the distribution equity of mental health institutions and doctors is both higher than that of beds and nurses. As for geographical allocation of mental health resources, the Theil index of mental health institutions, beds, doctors and nurses of Hunan in 2018 is 0.04, 0.11, 0.07 and 0.10 respectively. Particularly, the Theil index minimizes with mental health institutions, indicating the highest equity, and maximizes with bed allocation, indicating the lowest equity. The Theil index of four indicators (mental health institutions, beds, doctors, nurses) of Hunan in 2018 is generally similar to the situation reflected by Gini index, as the equity of medical health institution and doctor distributions is higher than that of beds and nurses, and the equity of population-based allocation is higher than that of geographical allocation (Table 6).

**Table 6:** The Theil index of resource allocation of mental health institutions.

Resource category	Population allocation	Geographical allocation
Mental health institution	0.01	0.04
Beds	0.04	0.11
Doctors	0.01	0.07
Nurse	0.04	0.10

## Discussion

The high incidence rates, long therapeutic period, and high recurrence severely impact the physical and mental health of patients, which have caused huge influence and burdens to individuals, families and the society. A study on diseases-caused burdens in China demonstrated that mental and behavioral disorders accounted for 9.5% of disability adjusted life years and 23.6% of years lived with disability [26]. The incidence rate of mental disorders among Chinese adults in 2009 was 17.5% [27–29]. The burdens of mental disorders and medical staff abuse in 2013 accounted for 10% of total burden of diseases in China and for 17% of total burden of diseases worldwide [30]. In the face of the increasing prevalence of mental disorders and the growing demand for mental health services, understanding the present situation and equity of mental health resource distribution is urgent, which is of great significance to further optimize the configuration of mental health resources.

### Shortage of mental health resources and its low utilization

In 2018, there were 141 mental health institutions in Hunan Province, with the largest proportionof specialized mental hospitals (63.1%) and the lowest proportion of mental rehabilitation institutions (1.4%). By comparison, there were 877 general hospitals and 174 traditional Chinese medicine hospitals in Hunan in 2018, which were 6.2 and 1.2 times the number of mental health institutions respectively [31]. This comparison indicates the total number of mental health institutions is still low, and mental rehabilitation institutions are deficient, which is similar to the situations of mental health resources in other provinces of China. One reason may be that the serving target of mental health institutions is a niche market. Another reason is that financial input and profit from mental health institutions are significantly deficient compared with other health institutions, and the majority of expenses by mental health institutions come from government investment, and most of such institutions are the governments-built specialized mental hospitals.

In addition, based on the millions of mental health patients and its increasing rate in Hunan province [16,17,32], the mental health resources utilization was at a low level. Taking the total number of outpatient (1288047) and the daily number of outpatient services (1.70) for examples, the number of psychiatric outpatients and inpatients is much lower compared with the actual number of mental health patients in Hunan province. Similarly, compared with the number of beds in Hunan Province, the utilization of beds is lower. There were a total of 25554 public mental health beds in Hunan by the end of 2018, with 38911 beds actually opened, with a density of 5.31 beds/10000 people. The number of actual open beds was 1.5 times the number of public beds. These results indicated that many patients with mental disorders did not receive effective treatment, which were related to the insufficient utilization of resources and obviously lack of mental health resources.

### Distribution of mental health resources among Hunan province

From the perspective of regional distribution, mental health institution distribution of Hunan is differentiated obviously, as such institutions are concentrated in Changsha, Zhuzhou and Xiangtan, as the number of mental health institutions maximizes in Shaoyang, followed by Changsha, and minimizes in Zhangjiajie. The reason may be that Changsha, Zhuzhou and Xiangtan are economically prosperous with a huge population, and the governmental fiscal revenue and thereby fiscal investment to public health from Changsha are higher than that of Zhangjiajie. Moreover, large numbers of mental health workers and mental health institutions are migrating from rural poverty-stricken communities to richer urban communities in developing countries, which further aggravates the shortage of mental health care providers [33–36].

In terms of beds, the numbers of beds differed among cities in Hunan, as the maximum number of actual open beds (from Changsha) is 12.07 times the minimum number (from Zhangjiajie). The bed density is 5.31 beds/10000 in Hunan, and varies between 3.60 and 12.10 beds/10000 among cities, showing large differences in bed allocation. The largest bed density (from Changsha) is 3.8 times the smallest level (from Yongzhou). The bed density of Hunan is higher than the global average level (4.3 beds/10000), but is still largely lower compared with developed countries. World Mental Health Atlas 2017 demonstrates that the average number of mental health beds in developed countries is 7.13 beds/10000, and bed configuration ways are diverse [37]. Additionally, though the bed numbers in most cities of Hunan have reached the provided standard in China, the bed numbers of certain cities are still far below the global average level.

Considering the long and periodic treatment of mental disorders patients and the trend of de institutionalized rehabilitation treatment, a mental health network of the province-city-county-township/community covering urban and rural areas has been gradually established in Hunan province since the implementation of the 686 Project. However, the mental health workers and the development of mental health institutions are still insufficient for low welfare, social status, work prospects and other reasons [38]. So we suggest that local governments should enlarge investment for human resources and institutions, and gradually strengthen early screening and diagnosis of mental diseases so as to achieve early discovery, early diagnosis and early treatment and reduce the demand for hospitalization. Furthermore, construction of rehabilitation and community resources should be strengthened to promote hospital turnover, reduce the demand for in-hospital beds and further improve service efficiency.

### Allocation of mental health human resources

By the end of 2018, the density of mental health doctors in Hunan is higher than, and the density of mental health nurses is lower than the world average levels, and particularly, the doctor densities of Changde, Yongzhou and Zhangjiajie are below the world average level. The doctor to nurse ratio in Hunan is higher than and the bed to nurse ratio is lower than the global average levels, but are both far lower than the levels in developed countries [39]. Moreover, the numbers of mental therapists, mental consultants, rehabilitation therapists, social workers, and public health doctors are still far deficient. One possible reason for such a phenomenon is that the recognition of mental health jobs is very low among the public, and the majority of mental health workers come from professionals, but the culture period of one mental health doctor is very long, and the incomes of mental health workers are lower than medical staff from other fields. Another reason may be related to the feeling of satisfaction with mental health jobs. Reportedly, the pay and reward are far below the expectations of mental health workers, so mental health workers are rather deficient and are mainly medical staff [40]. We suggest increasing the number of mental health workers through talent introduction or oriented training, or using multi-point practice to expand the mental health workers from Changsha to alleviate the shortage of mental health workers.

### Situations of diagnosis and treatment

The number of annual outpatient services by mental health institutions in Hunan in 2018 was 1288047. The number of daily outpatient services maximized in Zhuzhou, followed by Changsha, and minimized in Yongzhou, which may be related to the unevenness of economic development and medical resource distribution among cities in Hunan. Doctors in developed areas such as Changsha bear a heavy burden. In economy-stricken areas (e.g. Yongzhou), the burdens on doctors are gentle, and despite the doctor densities are above the global average levels, the medical staff still cannot meet social demands, which caused the large numbers of outpatient services and in-hospital patients. The possible cause is the gathering of patients from other places of Hunan to Changsha and other cities with huge populations and higher-quality medical services. We suggest further reinforcing the execution of graded diagnosis and treatment while increasing the numbers of medical staff, and to diverse patients of mental diseases (e.g. relieving the gathering of patients in certain cities).

### Equity of mental health resource allocation

The allocation of mental health resources in Hunan is very balanced, but it still needs to be further improved. The Gini index of population-based allocation shows that distributions of doctors and institutions are highly equal, and so do the distributions of nurses and beds. Nevertheless, allocations of nurses and beds still needs to be optimized. As for equity of geographical allocation, mental health resources are concentrated in developed areas, especially Changsha, which ranks the first in the numbers of institutions, beds, doctors and nurses per square kilometers. Comparison between Gini index and Theil index indicates mental health resource allocation based on population is more equitable than that of geographical allocation. One reason is that allocation of health resources in China has been based on the population of jurisdiction for a long time, but geographical factors are rarely considered. Moreover, the excessive elasticity of health investment policies in China is another primary cause for the spatial inequity of mental health resource allocation. The increasing rate of medical health financial investment from governments is slower than the increasing rate of fiscal spending, so the proportion of health investment gradually declines, leading to the shortage of mental health resources. Additionally, the input and output of hospitals in economically developed areas are larger, so these areas possess stronger in-system discourse power and can acquire more mental health resources, which further decreases the resources in less-developed areas. Consequently, inequity of regional allocation and gaps of mental health resources among cities are further enlarged. Patients from less-developed areas are under relatively poor economic conditions and less aware of mental diseases, and feel more ashamed of diseases, which reduce the utilization rate of mental health services. As a result, the self-compensation ability of mental health institutions is decreased, which prevents further development and enlarges the gaps with economically developed areas.

## Conclusion

The shortage of mental health resources, especially the shortage of human resources, is still the priority issue for policy-makers to increase and optimize in Hunan in the future. Moreover, the utilization of mental health resources was low, although its equity was fair. Policy-makers need to consider the high utilization and geographical accessibility of health resources among different regions to ensure people in different regions could get access to available health services.
